# Case report: lupus nephritis with autoantibodies to complement alternative pathway proteins and C3 gene mutation

**DOI:** 10.1186/s12882-015-0032-6

**Published:** 2015-03-30

**Authors:** Pilar Nozal, Sofía Garrido, Jorge Martínez-Ara, María Luz Picazo, Laura Yébenes, Rita Álvarez-Doforno, Sheila Pinto, Santiago Rodríguez de Córdoba, Margarita López-Trascasa

**Affiliations:** Immunology Unit, University Hospital La Paz, IdiPAZ, Madrid, Spain; Unit 754, Center for Biomedical Research on Rare Diseases (CIBERER), Madrid, Spain; Department of Nephrology, University Hospital La Paz, IdiPAZ, Madrid, Spain; Department of Pathological Anatomy, University Hospital, IdiPAZ, Madrid, Spain; Biological Research Center, CSIC, Madrid, Spain; Unit 738, Center for Biomedical Research on Rare Diseases (CIBERER), Madrid, Spain

**Keywords:** Complement alternative pathway, Autoantibodies, Complement C3, Lupus nephritis

## Abstract

**Background:**

Glomerulonephritis is one of the most severe complications of lupus, a systemic disease with multi-organ involvement, with tissue damage produced mainly by complement activation. As a result of this activation, patients with active lupus present hypocomplementemia during disease flares, but C3 and C4 levels are recovered between episodes.

**Case presentation:**

We present a patient who suffered two lupus nephritis episodes in 5 years, achieving complete remission with treatment after both of them, but with C3 levels persistently below normal range. Genetic study revealed that the patient carried a mutation in heterozygosis in the C3 gene. Serial sera samples were analyzed, and autoantibodies to complement alternative pathway proteins (Factor I, Factor B, C3 and Properdin) were found. Functional assays showed that these autoantibodies cause alternative pathway activation.

**Conclusion:**

This case is the first reported of a heterozygous C3 mutation associated with lupus nephritis and autoantibodies against complement alternative pathway proteins (Factor I, Factor B, C3 and Properdin).These autoantibodies cause activation of this pathway and this fact could explain that the tissue damage is restricted to the kidney.

**Electronic supplementary material:**

The online version of this article (doi:10.1186/s12882-015-0032-6) contains supplementary material, which is available to authorized users.

## Background

Lupus patients usually have low C3 and C4 levels during disease flares because of activation of the complement system by immune complexes (ICs) [1]. However, in remission periods, complement levels reach normal values. Activation of complement system by deposited ICs cause tissue damage, but, in the other hand, components of the classical pathway (CP) have a protective role in facilitating the clearance of ICs and apoptotic bodies, and their deficiency increases the risk of SLE [2,3], this association explained not only by ICs removal from circulation, but also by other mechanisms, including reduction of activation thresholds of lymphocytes and loss of self-tolerance [4,5].

## Case presentation

**Figure 1:**
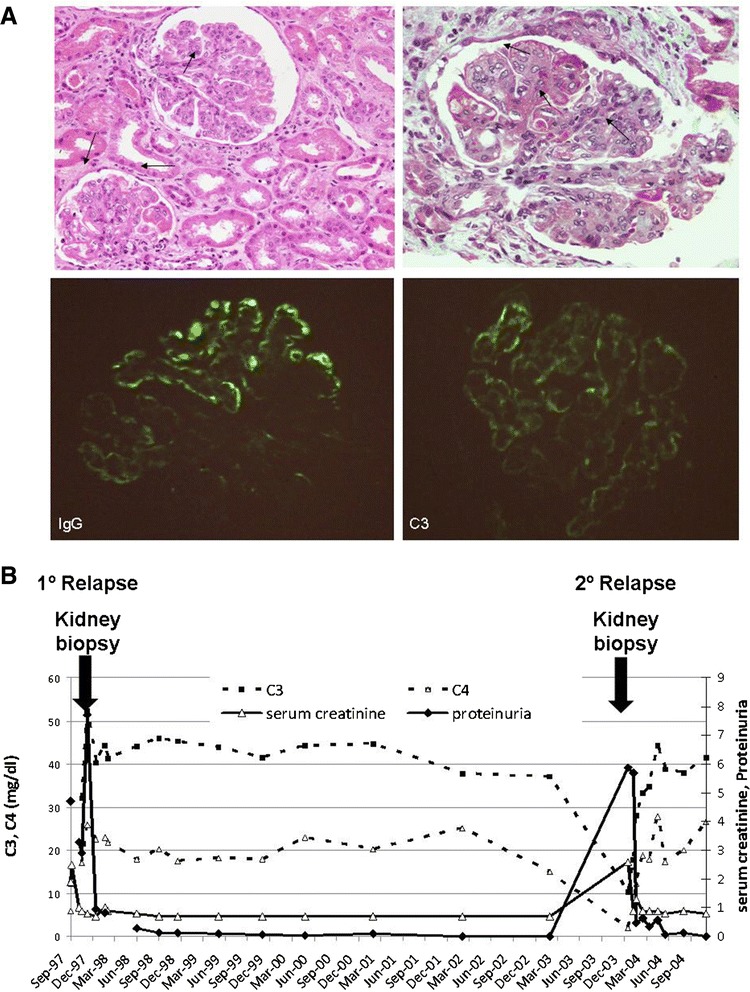
**First kidney biopsy images, and renal function and complement evolution over 7 years. A)** Light microscopy showing a glomerulus with endocapillary hipercellularity and karyorrhexis [hematoxylin-eosin (HE)], upper left panel. Light microscopy showing glomerular subendothelial deposits with wire loops lesions (masson trichrome), upper right panel. Immunoflourescence micrograph showing granular IgG deposits in the glomerular capillary walls, in a diffuse and global distribution, bottom left panel. Immunoflourescence micrograph showing subendothelial deposits of C3, bottom right panel. **B)** Seven year evolution of C3 and C4 levels, serum creatinine and proteinuria. Arrows indicate hospitalizations and renal biopsies due to nephrotic syndrome and renal insufficiency.

In 1997, a 20 year-old woman was admitted to the hospital due to renal insufficiency and nephrotic syndrome. She presented lower extremity oedema and high blood pressure, but no articular or cutaneous manifestations. Laboratory analysis showed serum creatinine 1.9 mg/dl, serum albumin 2.2 g/dl and proteinuria 4.7 g/24 h with hematuria. C3 and C4 levels were reduced (12.2 and 5.9 mg/dl, respectively), anti-nuclear and anti-DNA autoantibodies were positive. Other autoantibodies, including anti-GBM and C3NeF, were negative. A renal biopsy showed generalized and diffuse glomerular involvement, endocapillary hypercellularity with luminal occlusion and hyaline thrombi. Moderate mesangial proliferation in addition to acute inflammatory infiltrate was observed. Subendothelial deposits with wire loop images were present. On direct immunoflourescence, irregular deposits of C3, C1q, IgM, IgG and IgA were evident in the capillary walls and mesangium. The patient was diagnosed with active, diffuse, global, proliferative glomerulonephritis class IV-G lupus nephritis (Figure 1A).

She was treated with intravenous steroids plus oral prednisone with progressive reduction, and cyclophosphamide pulses for one year, with dosage adjustments. Renal function improved gradually, and one year after hospitalization, the patient presented complete remission and treatment was stopped.

She remained in clinical and analytical complete remission for five years, with the exception of C3 levels that were persistently below the normal range. In 2003 she was hospitalized again with renal insufficiency and nephrotic syndrome, with proteinuria 5.9 g/24 h, serum creatinine 2.2 mg/dl, C3 10.3 and C4 1.8 mg/dl. A new renal biopsy showed similar findings than the previous one, with signs of chronic activity.

She recovered renal function in 5 months receiving analogous treatment regimen as initially, with negative proteinuria and analytical parameters normalized with the only exception of C3 levels (evolution over 7 years is shown in Figure 1B). Thus, C3 levels were measured in her living family members, and her mother was found to have reduced C3 levels too.

In 2013, under informed consent, the patient was studied in attempt to characterize possible alterations in complement alternative pathway (AP), searching for either mutations or autoantibodies that caused these decreased serum C3 levels (methods are described in Additional file 1: Methods). The genetic study revealed that the patient and her mother carried a mutation in heterozygosis in exon 2 of C3 gene (c.131_146del; p.Leu44Argfs*19). This mutation generates a premature stop, and is thought to generate a truncated non-functional protein.

**Figure 2:**
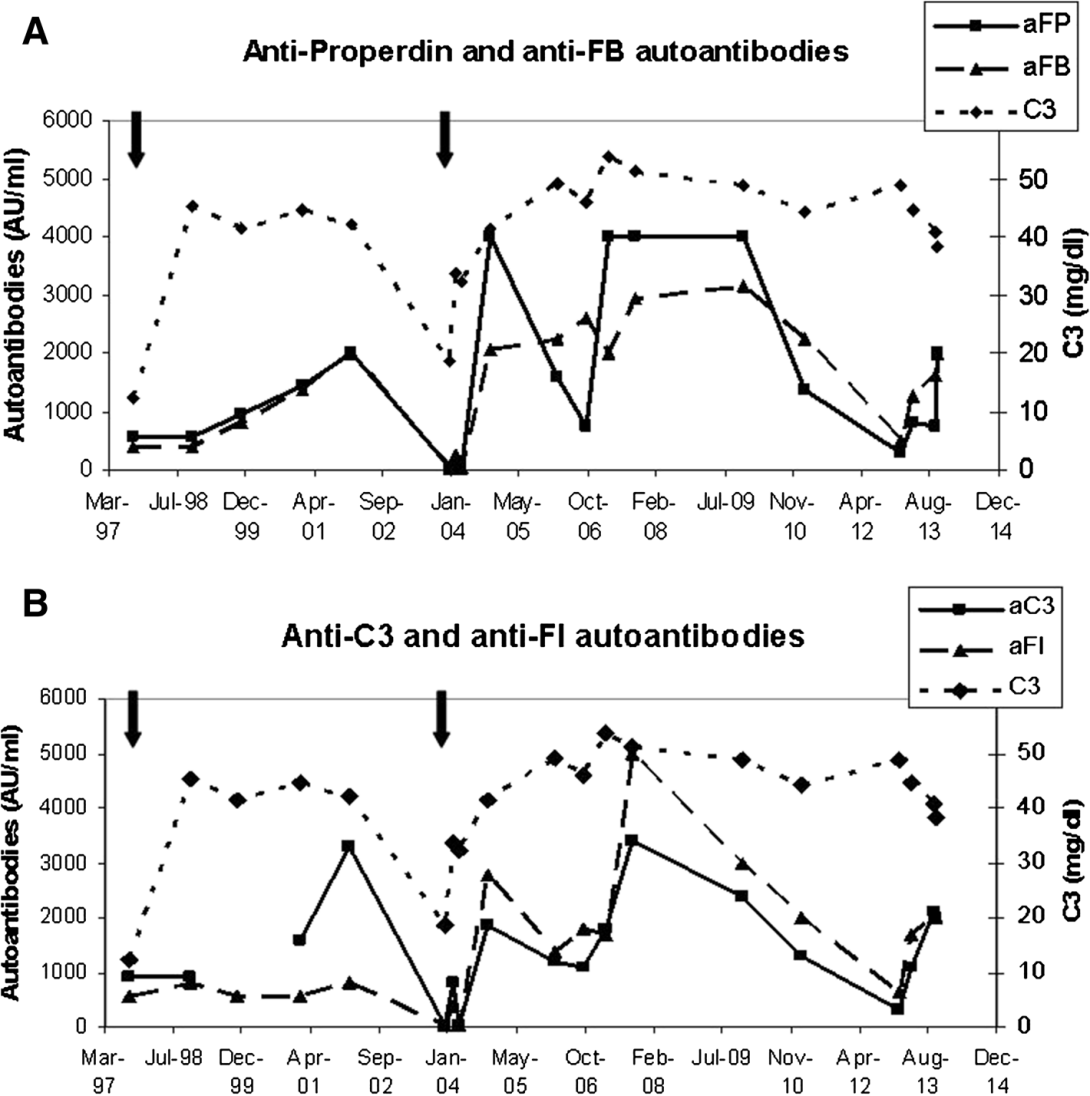
**C3 levels and autoantibodies titers follow up for a 16-year period.** In **A**, anti-FB and anti-properdin autoantibodies are shown along with C3 levels, and in **B**, anti-FI and anti-C3. Black arrows indicate hospitalizations due to renal insufficiency and the time when renal biopsies were performed. C3 normal range: 75–150 mg/dl.

In addition to the mutation, autoantibodies against C3, complement Factor B (FB), Properdin and Factor I (FI) were detected in the patient’s serum, but not in her mother. Serial sera samples were recovered and autoantibodies were retrospectively tested along a period of 16 years (Figure 2).

Autoantibodies to FI, C3, FB and Properdin were detected in nearly all patient’s studied samples, as well as significant levels of circulating complexes of IgG with FB and Properdin (Additional file 2). During her second hospitalization, the autoantibodies became undetectable and remained this way for at least 4 months, probably as an effect of reduced IgG levels (440 mg/dl) due to high proteinuria and the immunosuppressive treatment received. After that, autoantibodies reached high titers again, but no relapse ocurred, with only low doses of hydroxychloroquine as treatment.

Despite carrying the same mutation that her mother, C3 levels of the patient were always lower than hers, so functional studies were carried out to determine whether autoantibodies against AP proteins were responsible for this additional C3 reduction.

Assays were designed to evaluate the capacity of these antibodies to activate AP, both in fluid phase and on surfaces. In AP-50 hemolytic assays, purified IgGs from the patient reduced lysis drastically when preincubated with NHS, but lysis was restored when more NHS was added along with rabbit erythrocytes. This restoration correlated with AP activation in the fluid phase, as observed by C3 reduction when measured by nephelometry following the incubation of NHS with patient’s IgG. Nephelometric C3 and C4 measures revealed that autoantibodies caused a 10% reduction in C3, while maintaining normal C4 levels for NHS. IgG purified from pooled normal human serum had no effect on the C3 and C4 measures (data not shown). This C3 reduction from NHS in fluid phase was analyzed by western blot, showing proteolytic cleavage of NHS C3 induced by patient’s IgG (Additional file 3).

These assays revealed that the autoantibodies against AP proteins cause activation of this pathway only in fluid phase. This fact, in addition to the C3 mutation, may be responsible for the reduced C3 levels present in this patient and the limited renal damage.

## Conclusion

One of the main functions of complement system is recognition and elimination of pathogens, but it is also involved in clearance of ICs and cellular debris, in the acquisition of self-tolerance and in modulation of the adaptive immune response [4,6]. Complement activation mediates inflammation and tissue damage in autoimmune diseases, but also has a protective role, as complete deficiencies of CP components are associated with SLE [2,3]. C3 deficiencies are extremely infrequent, but patients usually suffer from repeated infections, and in some cases, immune complexes-related disorders are reported [3,6,7]. Even though SLE is an immune complexes disease, its association with homozygous C3 deficiency has only been reported in 3 Japanese patients [8], and so far, partial deficiencies of complement proteins have not been associated with SLE [5,6].

Reduced C3 levels due to the mutation in this patient could favour the appearance of autoimmunity, not only due to C3 role in self-tolerance development, but also because of defective immune complex clearance caused by hypocomplementemia. Furthermore, autoantibodies to AP proteins induce an additional reduction in serum C3 increasing deposition of complement activation products in renal glomeruli and contributing to local inflammation and tissue injury [9].

In immune complexes-mediated diseases, organ damage is caused by deposition of these circulating complexes and complement activation through classical pathway. This patient’s autoantibodies recognizing AP proteins might accumulate in the glomerulus as ICs and activate AP locally, exacerbating renal injury and contributing to disease pathogenesis.

Furthermore, in this patient, complement activation in fluid phase induced by the continuous presence of autoantibodies against AP components may play role in her serum complement profile with low C3 solely, and the absence of lesions in other organs apart from kidneys. Restricted renal damage also occurs in other pathologies caused by deregulated systemic complement activation, such as Dense Deposit Disease. Moreover, in a lupus nephritis model (MRL-lpr mice), in the absence of factor H (which leads to uncontrolled AP activation) disease progression is accelerated [10]. This outcome supports the pathogenic role of complement AP activation in immune complex-mediated diseases [2,10].

To our knowledge, this is the first reported case of a heterozygous mutation in the C3 gene, clinically presenting as lupus nephritis, with autoantibodies against complement alternative pathway proteins that induce complement activation, and this fact could explain the tissue damage is restricted to the kidneys.

## Consent

Written informed consent was obtained from the patient for publication of this Case report and any accompanying images. A copy of the written consent is available for review by the Editor of this journal.

## Additional files

Additional file 1:
**Methods.**
Additional file 2:
**Evolution of circulating immunocomplexes of IgG with properdin or FB and respective autoantibody titers in the studied sera samples.** Sera from 14 healthy controls were assayed for circulating immunocomplexes, and the mean OD and standard deviation from these samples was calculated. Arbitrary values were assigned in relation with this calculated mean OD, considering positive values those that were above the mean plus 2 standard deviations from the controls. Additional file 3:
**Alternative pathway activation caused by patient’s IgG.** A) Effect of IgG on total complement activity, measured in a hemolytic AP50 assay with rabbit erythrocytes. Purified IgGs from the patient, from pooled NHS and from a C3NeF patient (from 2 μg/μl to 0.125 μg/μl) were preincubated with NHS in a VBS-EGTA containing MgCl_2_ before addition of rabbit erythrocytes. B) Scheme of C3 proteolysis during complement activation. C3 protein is composed of α and β chains. When complement is activated, C3 convertase releases C3a from the α chain while β chain remains intact. Subsequent proteolytic fragments of the α chain are generated by FI to inactivate C3b. C) C3 proteolysis induced by patient’s IgG. Purified IgG from the patient or from pooled NHS was incubated with NHS in EDTA (no complement activation) or EGTA-Mg buffer (alternative pathway activation), C3 cleavage in the fluid phase was analyzed by WB. D) Quantitative analysis of C3 cleavage. C3 bands from western-blot experiments were densitometred, and normalized using β chain intensity. The percentage of variation between EDTA and EGTA buffer was calculated for each band, considering EDTA samples as 100%. The graphic represents the mean variation of intensity calculated from 6 experiments.

## Abbreviations

SLE: Systemic lupus erythematousus
ICs: Immunocomplexes
CP: Complement classical pathway
AP: Complement alternative pathway
FB: Factor B
FI: Factor I
