# Supplementary material for: Case report: lupus nephritis with autoantibodies to complement alternative pathway proteins and C3 gene mutation
Source: BMC Nephrol. 2015 Mar 30;16:40. doi: 10.1186/s12882-015-0032-6 (PMC4415395; doi:10.1186/s12882-015-0032-6)
Supplement: Additional file 1: — Methods. [file 12882_2015_32_MOESM1_ESM.doc]

**Supplementary material:**

**Methods**

Autoantibodies to AP protein screening: ELISA plates were coated with 100 ng/well of purified FB, FI, C3 or Properdin (Quidel and/or Comptech). Serum samples were diluted in PBS-BSA 0.1%, and binding of autoantibodies was detected with polyclonal anti-human IgG-HRP conjugated antibody (Jackson) in ABTS substrate.

Presence of circulating complexes of IgG with FB and IgG with Properdin in serum: to detect IgG bound to target protein, sera from patients and healthy controls were incubated on ELISA plates coated with 100 ng/well of monoclonal anti-Properdin or anti-FB antibodies (Quidel), and anti-human IgG-HRP was used to detect circulating complexes (Fig S1). Significant levels of these circulating complexes were detected in the patient’s sera samples with positive autoantibodies, but not in healthy controls nor in C3NeF-positive patients.

Functional studies were designed to evaluate autoantibodies effect on alternative pathway. To that end, fluid phase and hemolytic assays were performed with purified patient’s IgG.

IgG fraction was purified from the patient’s and normal human sera by anion exchange chromatography on DEAE-Sephacel columns in 20mM phosphate buffer pH 7.5. More of the 80% of the IgG was recovered on the excluded fractions. Purified IgGs were tested simultaneously in the ELISA assay with their respective sera, diluting them to the same concentration.

The effect on total complement activity was measured with an AP-50 assay. Purified IgGs (from 2 µg/µl to 0.125 µg/µl) were preincubated with NHS for 15 min at 37°C in AP buffer (VBS-EGTA containing MgCl_2_). Rabbit erythrocytes (10^7^cells/sample) were added, and samples were incubated again for 15 min at 37ºC. The reaction was stopped with VBS-EDTA and supernatant absorbance was measured at 412nm after centrifugation. The percentage of lysis was calculated using a total lysis control. In these hemolytic assays, the patient’s IgG reduced lysis when preincubated with NHS, but lysis was restored when more NHS was added along with rabbit erythrocytes. Because of this C3 reduction observed when NHS was incubated with patient’s IgG, C3 proteolytic cleavage was analyzed by western blot. For that purpose, IgGs were incubated with NHS in EDTA or EGTA-MgSO_4_ containing buffer for 1.5 h at 37°C. A total of 2µl of each sample was loaded in SDS-PAGE 12% gel, and then transferred to a PDVF membrane. WB membrane was blocked and then incubated with goat polyclonal anti-C3 antibody (Fig S2).

**Supplementary figure legends:**

Supplementary figure 1: Evolution of circulating immunocomplexes of IgG with properdin or FB and respective autoantibody titers in the studied sera samples. Sera from 14 healthy controls were assayed for circulating immunocomplexes, and the mean OD and standard deviation from these samples was calculated. Arbitrary values were assigned in relation with this calculated mean OD, considering positive values those that were above the mean plus 2 standard deviations from the controls.

Supplementary Figure 2: Alternative pathway activation caused by patient’s IgG.

A) Effect of IgG on total complement activity, measured in a hemolytic AP50 assay with rabbit erythrocytes. Purified IgGs from the patient, from pooled NHS and from a C3NeF patient (from 2µg/µl to 0.125µg/µl) were preincubated with NHS in VBS-EGTA containing MgCl_2_ before addition of rabbit erythrocytes.

B) Scheme of C3 proteolysis during complement activation. C3 protein is composed of α and β chains. When complement is activated, C3 convertase releases C3a from the α chain while β chain remains intact. Subsequent proteolytic fragments of the α chain are generated by FI to inactivate C3b.

C) C3 proteolysis induced by patient’s IgG. Purified IgG from the patient or from pooled NHS was incubated with NHS in EDTA (no complement activation) or EGTA-Mg buffer (alternative pathway activation), C3 cleavage in the fluid phase was analyzed by WB.

D) Quantitative analysis of C3 cleavage. C3 bands from western-blot experiments were densitometred, and normalized using β chain intensity. The percentage of variation between EDTA and EGTA buffer was calculated for each band, considering EDTA samples as 100%. The graphic represents the mean variation of intensity calculated from 6 experiments.
