# Supplementary figures and images for: Case report: lupus nephritis with autoantibodies to complement alternative pathway proteins and C3 gene mutation
Source: BMC Nephrol. 2015 Mar 30;16:40. doi: 10.1186/s12882-015-0032-6 (PMC4415395; doi:10.1186/s12882-015-0032-6)

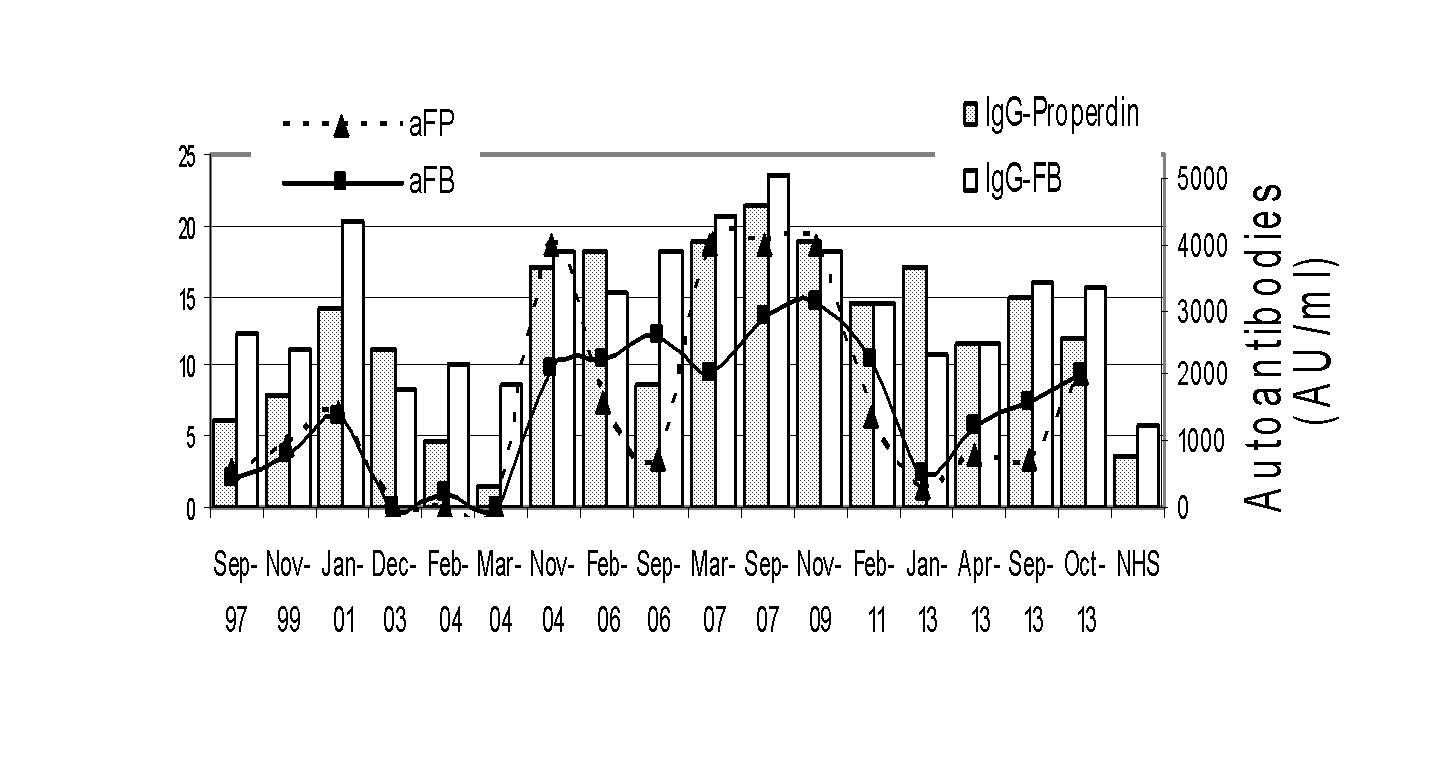

Supplement: Additional file 2: — Evolution of circulating immunocomplexes of IgG with properdin or FB and respective autoantibody titers in the studied sera samples. Sera from 14 healthy controls were assayed for circulating immunocomplexes, and the mean OD and standard deviation from these samples was calculated. Arbitrary values were assigned in relation with this calculated mean OD, considering positive values those that were above the mean plus 2 standard deviations from the controls. [file 12882_2015_32_MOESM2_ESM.jpeg]

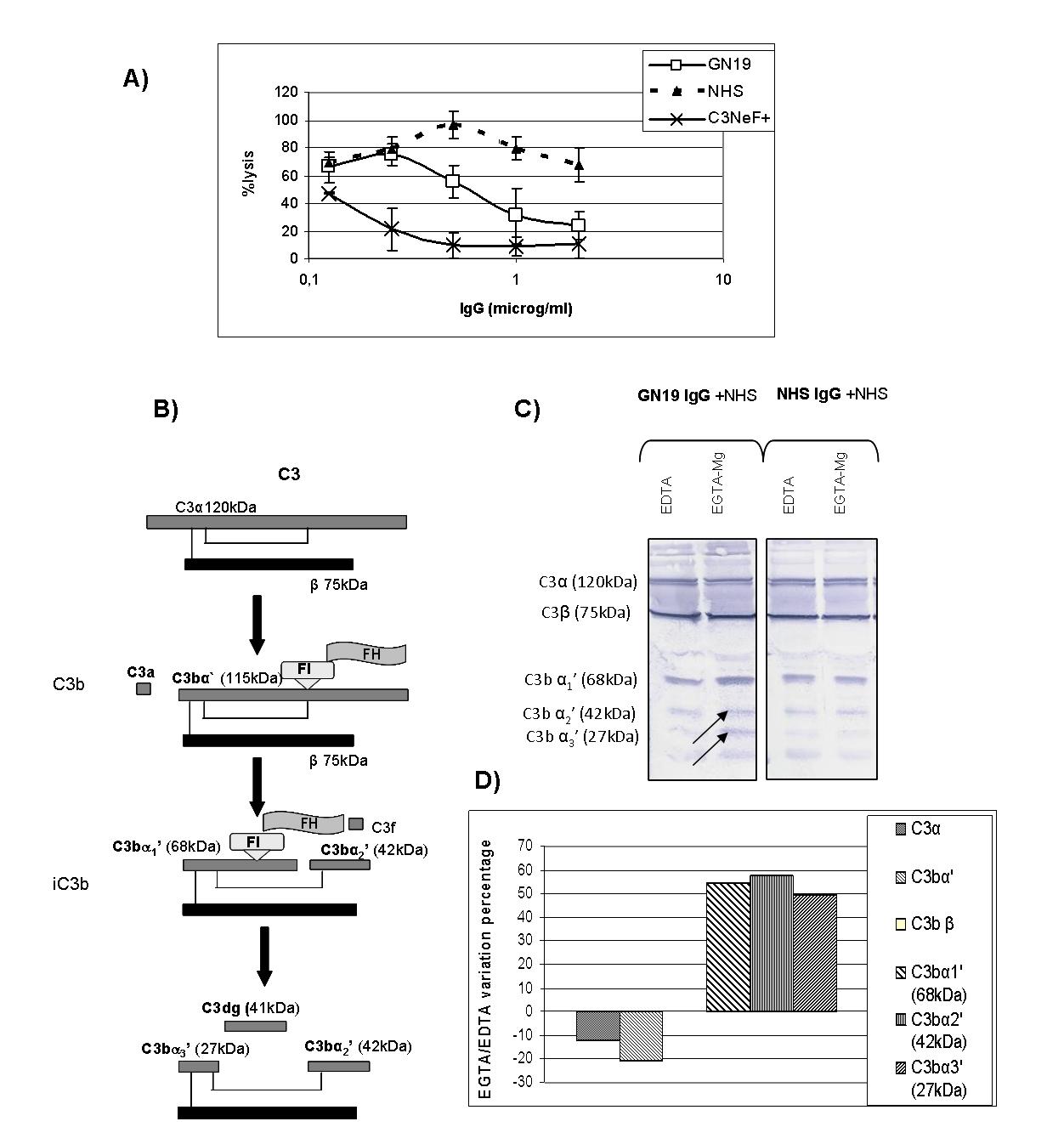

Supplement: Additional file 3: — Alternative pathway activation caused by patient’s IgG. A) Effect of IgG on total complement activity, measured in a hemolytic AP50 assay with rabbit erythrocytes. Purified IgGs from the patient, from pooled NHS and from a C3NeF patient (from 2 μg/μl to 0.125 μg/μl) were preincubated with NHS in a VBS-EGTA containing MgCl2 before addition of rabbit erythrocytes. B) Scheme of C3 proteolysis during complement activation. C3 protein is composed of α and β chains. When complement is activated, C3 convertase releases C3a from the α chain while β chain remains intact. Subsequent proteolytic fragments of the α chain are generated by FI to inactivate C3b. C) C3 proteolysis induced by patient’s IgG. Purified IgG from the patient or from pooled NHS was incubated with NHS in EDTA (no complement activation) or EGTA-Mg buffer (alternative pathway activation), C3 cleavage in the fluid phase was analyzed by WB. D) Quantitative analysis of C3 cleavage. C3 bands from western-blot experiments were densitometred, and normalized using β chain intensity. The percentage of variation between EDTA and EGTA buffer was calculated for each band, considering EDTA samples as 100%. The graphic represents the mean variation of intensity calculated from 6 experiments. [file 12882_2015_32_MOESM3_ESM.jpeg]
